# The Effect of the Clenbuterol—β2-Adrenergic Receptor Agonist on the Peripheral Blood Mononuclear Cells Proliferation, Phenotype, Functions, and Reactive Oxygen Species Production in Race Horses In Vitro

**DOI:** 10.3390/cells10040936

**Published:** 2021-04-17

**Authors:** Olga Witkowska-Piłaszewicz, Rafał Pingwara, Jarosław Szczepaniak, Anna Winnicka

**Affiliations:** 1 Department of Pathology and Veterinary Diagnostics, Institute of Veterinary Medicine, Warsaw University of Life Science—SGGW, 02-787 Warsaw, Poland; anna_winnicka@sggw.edu.pl; 2 Department of Physiological Sciences, Institute of Veterinary Medicine, Warsaw University of Life Sciences—SGGW, 02-787 Warsaw, Poland; rafal_pingwara@sggw.edu.pl; 3 Department of Nanobiotechnology, Institute of Biology, Warsaw University of Life Sciences—SGGW, 02-787 Warsaw, Poland; jaroslaw_szczepaniak@sggw.edu.pl

**Keywords:** exercise, thoroughbred, interleukin, proliferation, lymphocyte, monocyte, ROS, doping, cytokine

## Abstract

Clenbuterol, the β2-adrenoceptor agonist, is gaining growing popularity because of its effects on weight loss (i.e., chemical liposuction). It is also popular in bodybuilding and professional sports, due to its effects that are similar to anabolic steroids. However, it is prohibited by anti-doping control. On the other hand, it is suggested that clenbuterol can inhibit the inflammatory process. The cells from 14 untrained and 14 well-trained race horses were collected after acute exercise and cultured with clenbuterol. The expressions of CD4, CD8, FoxP3, CD14, MHCII, and CD5 in PBMC, and reactive oxygen species (ROS) production, as well as cell proliferation, were evaluated by flow cytometry. In addition, IL-1β, IL-4, IL-6, IL-10, IL-17, INF-γ and TNF-α concentrations were evaluated by ELISA. β2-adrenoceptor stimulation leads to enhanced anti-inflammatory properties in well-trained horses, as do low doses in untrained animals. In contrast, higher clenbuterol doses create a pro-inflammatory environment in inexperienced horses. In conclusion, β2-adrenoceptor stimulation leads to a biphasic response. In addition, the immune cells are more sensitive to drug abuse in inexperienced individuals under physical training.

## 1. Introduction

Clenbuterol (CLEN) is a selective β2-adrenergic agonist (β2/β1 ratio = 4.0) [1]. In addition, it is lipid-soluble, and thus it crosses the blood–brain barrier [1,2]. The action of CLEN is caused by binding to β2-adrenoceptors (β2-ARs) and activating adenylyl cyclase. The activation of adenylyl cyclase leads to an increase in intracellular concentrations of cyclic adenosine monophosphate (cAMP) and ultimately the activation of protein kinase A (PKA). Compared with other β2-ARs agonists, CLEN has greater potency, an extended half-life (25–40 h), and is more readily absorbed (70–80%) from the gastrointestinal tract [2]. β2-ARs are located throughout the body, including in the heart, gastrointestinal tract, liver, uterus, blood vessels, sweat glands (in horses), fat, and skeletal muscles [3,4,5]. In addition, β2-ARs are localized also at bronchial level, and therefore one of the indications of CLEN is as a bronchodilator [3].

Furthermore, β2-ARs are expressed on immune cells such as B and T lymphocytes, neutrophils, mast cells, monocytes, and eosinophils, and as a consequence, they are regarded as the main mediators of the immune effects of adrenoreceptors agonists [6]. In addition, β2-ARs play a key role in the process of immunological imbalance [7]. Some studies suggest that β2-ARs agonists play an anti-inflammatory role by decreasing the proinflammatory cytokines (TNF-α and IL-6) levels after lipopolysaccharide (LPS) challenge in humans, as well as in horses [8,9]. Additionally, they may reduce oxidative stress by decreasing reactive oxygen species production (ROS).

CLEN is one of the most popular abused drugs for weight loss in amateur sportsmen [10]. It has hypertrophic, lipolytic, and dose-dependent anabolic effects [11]. Authorities in several countries have prohibited the use of CLEN due to its numerous adverse effects in humans, such as cardiomyopathy and acute hepatic injury [12,13]. Unfortunately, it is readily available through internet commerce, and has proliferated the trend of abuse among bodybuilders and people who want to slim down quickly. In horses, CLEN is already used in the treatment of respiratory disorders, including asthma [14,15]. However, it is also used due to its connection with performance enhancement and its high availability in equine sports. In addition, CLEN treatment reduces bone mineral density (BMD) and mechanical resistance [16]. While there are no reported data for horses regarding the effect of this drug on bone metabolism, the implications are that it may potentially lead to orthopedic problems, especially in young, growing horses. Thus, special attention to this animal’s welfare should be paid.

Obtaining the best sporting results is an issue of major importance in both human and animal athletes. Unfortunately, sometimes it is also connected with performance enhancement. In human athletes, the use of doping ranges from 5 to 31% [17]. In recent years, there has been a huge scandal connected with abused drugs in horses that won millions of dollars around the world, according to a New York Times publication [18]. Horse racing is a multi-million dollar industry, and according to The American Horse Council Foundation, more than 1.2 million horses are used for racing [19]. As such, it is no surprise that drugs would be used illegally to increase the chances of a horse winning. Drug testing in equine sports poses different challenges compared to that in humans [20]. The prohibition of certain drugs is mostly done to ensure the welfare of horses and riders, and then to protect the fair play rules and the integrity of the breeding industry. 

Exhaustive and prolonged exercise may have an immunosuppressor effect during the recovery period. The suppression of immunity is referred to as the “Open Window Period”, which continues, depending on the protocols, from 3 to 17 h after physical activity in humans [21] and horses [22]. It is particularly important in race horses because they start intensive training at a very young age (2 years), making them prone to overtraining, which may reduce the immune system’s functionality. It was documented that with training development, the immune system of the equine athlete adapts [23,24]. In highly trained horses, an anti-inflammatory status develops, whereas in young horses, pro-inflammatory reactions occur, which has been confirmed in race and endurance horses [23,24,25]. Currently, the available data reporting the impact of CLEN on immunity are still ambiguous. Additionally, there are no published data on the influence of CLEN on the equine immune system, especially when racehorses are using the highest possible dosages of this drug. Besides this, horse athletes provide an excellent parallel for exercise-based studies of humans [23,24]. Thus, the aim of the study was to determine the effect of CLEN in various concentrations on the peripheral blood mononuclear cells (PBMCs) isolated from healthy racehorses after strenuous exercise.

## 2. Materials and Methods

### 2.1. Animals and Blood Sampling

To analyze the influence of clenbuterol at different doses on immune cells in racehorses at various fitness levels, healthy 2–4-year-old racehorses of both genders (50% male, 50% female; total *n* = 28) were enrolled in the study. The first group consisted of well-trained (WT) thoroughbreds (*n* = 14, 3–4 years old, average: 3.14 ± 0.36) with a history of good performance during the previous training season. In the second group were untrained (UT) thoroughbreds (*n* = 14, 2–3 years old, average: 2.29 ± 0.47) at the beginning of their race training. The environmental conditions and training regimen were the same for both groups relative to training level. The animals were stabled and trained by one trainer. Clinical examinations (the heart rate, mucous membranes (color and moisture), capillary refill time, and dehydration (measured as the time it takes for a pinched skin fold over the point of the shoulder to flatten)) and basic blood hematological and biochemical tests were performed before and after training by a veterinary practitioner, and these revealed no clinical symptoms of diseases. The training session was performed on an 800 m sand track at the speed ≈ 800 m/min on the same day to avoid weather influence. For UT, it was the first training session with a gallop, and for WT horses it was the beginning of another training season. Blood samples were collected by a jugular venipuncture 30 min after the training session using a BD Vaccutainer system with heparin tubes for PBMCs isolation. All samplings were a part of standard veterinary diagnostic procedure, and were performed according to the Polish legal regulations [26] and the European directive EU/2010/63. Approval of the Local Commission for Ethics in Animal Experiments was not required. 

### 2.2. Clenbuterol

Clenbuterol hydrochloride (CLEN) (Sigma-Aldrich, Saint Louis, MO, USA) was dissolved in phosphate-buffered saline (Life Technologies, Bleiswijk, Netherlands) and then diluted in RPMI 1640 Medium with GlutaMAX™ (Gibco, Life Technologies, Bleiswijk, The Netherlands). Cells were cultured in three different concentrations (0.6, 1.0 and 1.6 ng/mL) because CLEN reaches a concentration of 0.6–1.6 ng/mL in the plasma of horses [1,27,28]. The control samples were derived from the same horses that were cultured without CLEN.

### 2.3. Cell isolation and Culture

PBMCs were isolated from the heparinized blood of all horses by density gradient centrifugation (SepMate™-Lymphoprep™ System, Cologne, Germany). According to manufacturer instruction the cells were centrifugated for 1200× *g* for 10 min. Then, cell cultures were performed in RPMI 1640 Medium with GlutaMAX™ (Gibco, Life Technologies, Bleiswijk, Netherlands) containing 10% heat-inactivated horse serum, penicillin (100 IU/mL), streptomycin (100 μg/mL), nonessential amino acids (1%), MEM vitamins (100 μM), sodium pyruvate (1 mM) and amphotericin B (1 μg/mL) (Gibco™, Life Technologies, Bleiswijk, The Netherlands), after twice washing in 2% BSA. A total of 4 × 10^6^ freshly isolated PBMC was cultured in the absence or presence of phytohemagglutinin (PHA) (Sigma-Aldrich, St. Louis, MO, USA; 5 μg/mL). After 24 h the cells were washed and recombinant equine IL-2 (R and D Systems, Abingdon, UK; 100 U/mL) was added, and then the cells were incubated for another 3 days. All cells were incubated at 37 °C with 5% CO_2_.

### 2.4. Cell Staining

Samples designated for the determination of the cell proliferation were supravitally stained with CellTrace™ Violet Cell Proliferation Kit (Life Technologies, Bleiswijk, The Netherlands) before culturing according to the manufacturer’s instructions.

After 4 days the production of reactive oxygen species by isolated PBMCs was measured using the CellRox (CR) Deep Red Assay Kit (Life Technologies, Paisley, Scotland) according to the manufacturer’s protocol. Tert-butyl hydroperoxide solution (TBHP) was used as an inducer of reactive oxygen species (ROS) production. To minimalize the risk of cell damage and to obtain adhesive cells, Corning^®^ Cellstripper^®^ Solution (Mediatech, Inc., Manassas, VA, USA) was used according to the manufacturer’s instructions. For the analysis of lymphocytes, only non-adherent cells were collected, and these were also used for the determination of the cell proliferation. PBMCs were characterized by checking the expression of the surface markers using equine-specific antibodies or with documented cross-reactivity (included in Table 1). The appropriate amount and concentration of each antibody was determined empirically to obtain optimal labeling results. The controls included unlabeled cells, and when necessary, the FMO (fluorescence minus one) and “switch-off” approach (SWOFF) controls were used. As serum is effective in blocking nonspecific mAb binding, the 10% BSA (15 min at 4 °C) before staining with antibodies was used. The cells were incubated with antibodies for 20 min at 4 °C in eBioscience™ Flow Cytometry Staining Buffer (Life Technologies, Bleiswijk, The Netherlands) in the dark. The cells were then washed twice with 2% BSA and resuspended in 200 μL flow cytometry staining buffer, and immediately introduced into the cytometer. For FoxP3 staining the eBioscience™ FoxP3/Transcription Factor Staining Buffer Set (Life Technologies, Bleiswijk, The Netherlands) was used according to the manufacturer’s protocol.

### 2.5. Flow Cytometry Analysis

The gating strategy was shown in the previous study [23]. Doublets were removed from the analysis by setting the gate on single cells on the FSC-area (FSC-A) vs. FSC-high (FSC-H) dot plot. Cell proliferation was calculated from singlets. Next, the lymphocytes or monocytes were gated based on FSC and SSC dot plots. Then, the gate included lymphocytes, and analyses of CD4+, CD8+, and FoxP3+ cells were performed. The second sample included CD5+, CD14+, MHCII+ cells, and the median fluorescence intensity (MFI) of ROS was calculated on that cell population.

Flow cytometric analysis was performed using a FACSCanto II flow cytometer and Kaluza 1.5 software (Beckman Coulter, Brea, CA, USA); 10,000 cells of each sample were acquired. Prior to multicolor staining, the compensation was set using single-positive cells for each color.

### 2.6. ELISA 

The concentrations of cytokines (IL-1β, IL-4, IL-6, IL-10, IL-17, INF-γ, TNF-α,) were determined by commercially available immunoenzymatic commercial assays dedicated to equine species (Cloud-Clone Corp., Katy, TX, USA). The absorbance was measured via a Multiscan Reader (Labsystem, Helsinki, Finland) using a Genesis V 3.00 software program.

### 2.7. Statistical Analysis

The statistical analysis was performed in Prism software, version 5.0 (GraphPad Software, San Diego, CA, USA). The UT and WT horses were analyzed independently. One-way ANOVA and Tukey’s HSD post hoc test were applied to determine the statistical significance of control cells (not CLEN-treated) and CLEN-treated cells between different concentrations of the drug. A *p*-value < 0.05 was regarded as significant, whereas a *p*-value < 0.01 and *p*-value < 0.001 were highly significant.

## 3. Results

### 3.1. Clenbuterol Enhances Lymphocyte Proliferation

For the assessment of the effect of CLEN on lymphocyte proliferation, Cell Trace Violet staining was used. The data obtained are inversely proportional to the proliferative activity because with each cell division, the fluorescence intensity of the dye becomes lower. The data are shown as the reciprocal of the MFI value (1/MFI).

**Figure 1:**
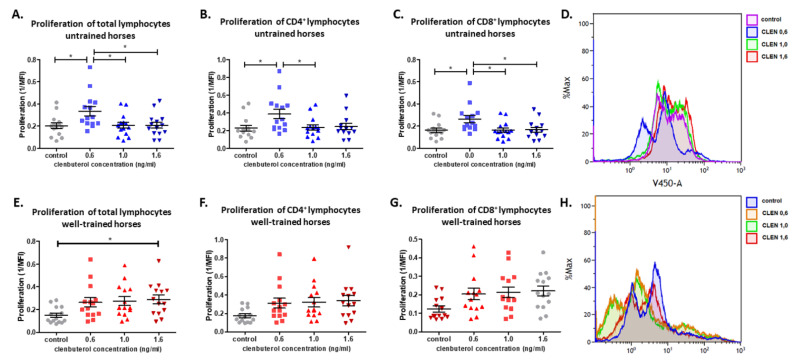
The graph shows total (**A**,**E**), CD4+ (**B**,**F**), and CD8+ (**C**,**G**) lymphocyte proliferation. Representative histogram showing proliferation of control and clenbuterol-treated lymphocytes harvested from untrained and well-trained horses. (**D**,**H**) Each dot represents one individual horse (*n* = 28) and means ± SEM (standard error of the mean) are presented. Significance levels are: * *p* < 0.05.

The study showed that CLEN enhances the proliferation of lymphocytes. A statistically significant increase in proliferation of lymphocytes obtained from untrained horses was observed under CLEN treatment at the lowest examined concentration (0.6 ng/mL) in comparison to non-treated cells. The changes in proliferation were observed in total lymphocytes, and both CD4+ and CD8+ cells. However, we did not observe an effect of CLEN at concentrations 1.0 and 1.6 ng/mL. Interestingly, we detected a statistical difference in T cell proliferation between cells treated with CLEN at 0.6, 1.0, and 1.6 ng/mL (Figure 1A–D). A significant difference between different CLEN concentrations also arose for CD4+ and CD8+. In the case of WT horses, a different trend in CLEN activity was observed. The increase in total lymphocyte proliferation was observed under CLEN treatment at the highest (1.6 ng/mL) concentration. We did not detect the influence of CLEN on lymphocyte proliferation in other examined concentrations. Furthermore, CLEN did not affect the CD4+ and CD8+ cells’ ability to proliferate (Figure 1E–H). 

### 3.2. Clenbuterol Influences on T Cell Phenotype

As was documented in a previous study, in horses, due to the lack of the equine-specific CD25 antibodies and the relatively low homology of the equine CD25 gene, CD4+FoxP3+ cells are also considered as Tregs and CD8+FoxP3+ cells as cytotoxic regulatory cells [23].

**Figure 2:**
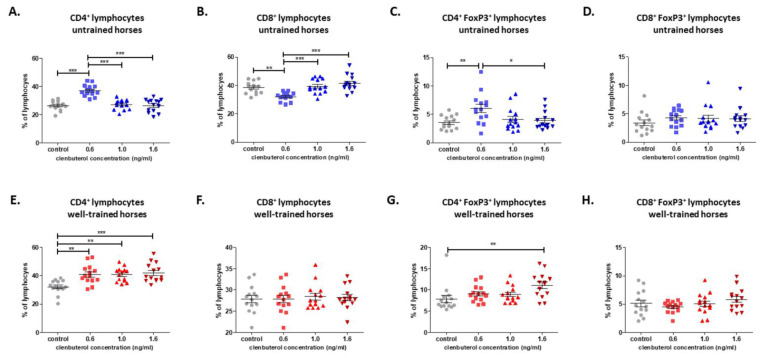
Graphic representation showing percentages of positive cells: CD4+ (**A**,**E**), CD8+ (**B**,**F**), CD4+FoxP3+ (**C**,**G**), and CD8+FoxP3+ (**H**,**D**) gated from total lymphocytes. Each dot represents one individual horse (*n* = 28) and means ± SEM (standard error of the mean) are presented. The significance levels are: * *p* < 0.05; ** *p* < 0.01, and *** *p* < 0.001.

The research demonstrated that CLEN supplementation (0.6 ng/mL) increased and decreased CD4+ and CD8+ cell percentage, respectively, in the culture of cells obtained from untrained horses. In the culture with CLEN at 1.0 and 1.6 ng/mL concentrations, this effect was not observed, and the cell percentage under these stimulations was at a similar level to that in the untreated group (Figure 2A,B). Moreover, a statistical difference in CD4+ and CD8+ was observed between cells treated with CLEN at 0.6 ng/mL and cells treated with CLEN at 1.0 and 1.6 ng/mL. Furthermore, we observed an increase in CD4+ FoxP3+ T regulatory cell number under CLEN treatment (0.6 ng/mL) in comparison to control cells and cells treated with 1.6ng/mL CLEN. The CD8+ FoxP3+ T regulatory cell number remained at the non-treated culture level (Figure 2C,D).

In the culture of cells harvested from WT horses, a higher percentage of CD4+ T cells in the group treated with CLEN (all examined concentrations) in comparison to non-treated cells (Figure 2A) was observed. In particular, an increase in CD4+ FoxP3+ T regulatory cells was observed in cultures treated with CLEN at a concentration of only 1.6 ng/mL (Figure 2C), whereas cell treatment with CLEN at all concentrations did not affect the CD8+ and CD8+FoxP3+ cell numbers (Figure 2B,D).

### 3.3. Clenbuterol Influences on Monocyte Phenotype

**Figure 3:**
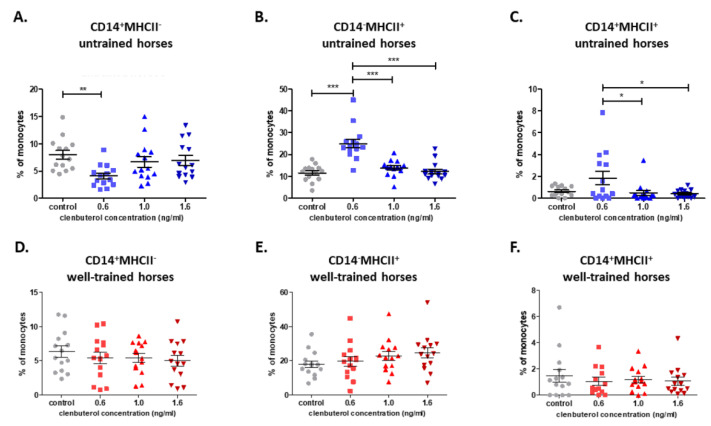
Graphic representation showing percentages of positive cells: CD14+MHCII− (**A**,**D**), CD14-MHCII+ (**B**,**E**), and CD14+MHCII+ (**C**,**F**) gated from total monocytes. Each dot represents one individual horse (*n* = 28) and means ± SEM are presented. The significance levels are: * *p* < 0.05; ** *p* < 0.01, and *** *p* < 0.001.

In cell cultures obtained from untrained horses, CLEN treatment at a concentration of 0.6 ng/mL decreased the CD14+MHCII- and increased the CD14-MHCII+ cell percentages (Figure 3A,B). Moreover, the number of CD14-MHCII+ cells was increased in the group treated with 0.6 ng/mL CLEN in comparison to the group treated with CLEN at higher concentrations (Figure 3B). An effect of CLEN on the CD14+MHCII+ cell number was not observed; however, the percentage of these cells was higher in culture with CLEN at 0.6 ng/mL in comparison to cultures with CLEN at 1.0 and 1.6 ng/mL concentrations (Figure 3D).

In well-trained horses, no effect of CLEN on the percentage of the subpopulation of monocytes was observed (Figure 3D–F).

### 3.4. Clenbuterol Decreased ROS Synthesis in Monocytes

**Figure 4:**
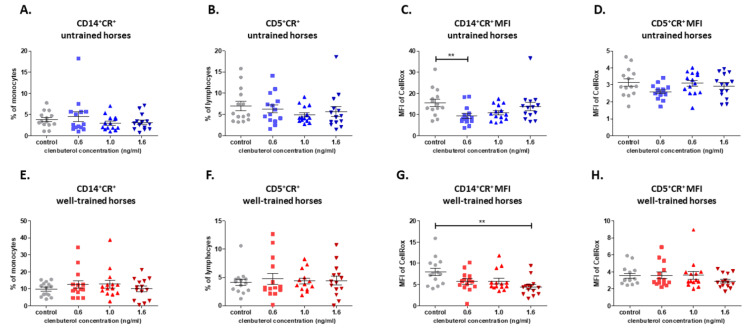
Graphic representation showing percentages of positive cells: CD14+CR+ (**A**,**E**), CD5+CR+ (**B**,**F**) gated from total lymphocytes/monocytes and median fluorescent intensity of CR (CellRox) in CD14+ (**C**,**G**) and CD5+ (**D**,**H**) cells. Each dot represents one individual horse (*n* = 28) and means ± SEM (standard error of the mean) are presented. Significance levels are: ** *p* < 0.01.

The examination of ROS production in monocytes showed that CLEN reduced this process. While a change in the percentage of cells that produced ROS under CLEN was not observed, the declined intensity of the ROS production, both in untrained (CLEN 0.6 ng/mL) and well-trained horses (CLEN 1.6 ng/mL) was confirmed (Figure 4A,C,E,G.)

In the case of lymphocytes, an influence of CLEN on the percentage of cells that produced ROS and on the intensity of this process was not demonstrated (Figure 4B,D,F,H.)

### 3.5. Clenbuterol Modulates Cytokine Production

In the PBMC of untrained horses treated with CLEN, higher secretions of IL-6 (0.6 ng/mL CLEN), IL-10 (0.6 ng/mL CLEN), and IFN-γ (1.6 ng/mL CLEN) in comparison to non-treated cells were observed (Figure 4C,D,F), while the secretion of IL-4 and TNF-α was inhibited by CLEN at the 0.6 ng/mL concentration (Figure 4B,G). No effect of CLEN was observed on the IL-1 and IL-17 production (Figure 4A,E). Additionally, a difference in cytokine (not IL-1β and IL-17) production by cells treated with the lowest (0.6 ng/mL) and cells treated with the highest (1.6 ng/mL) concentrations was observed. 

**Figure 5:**
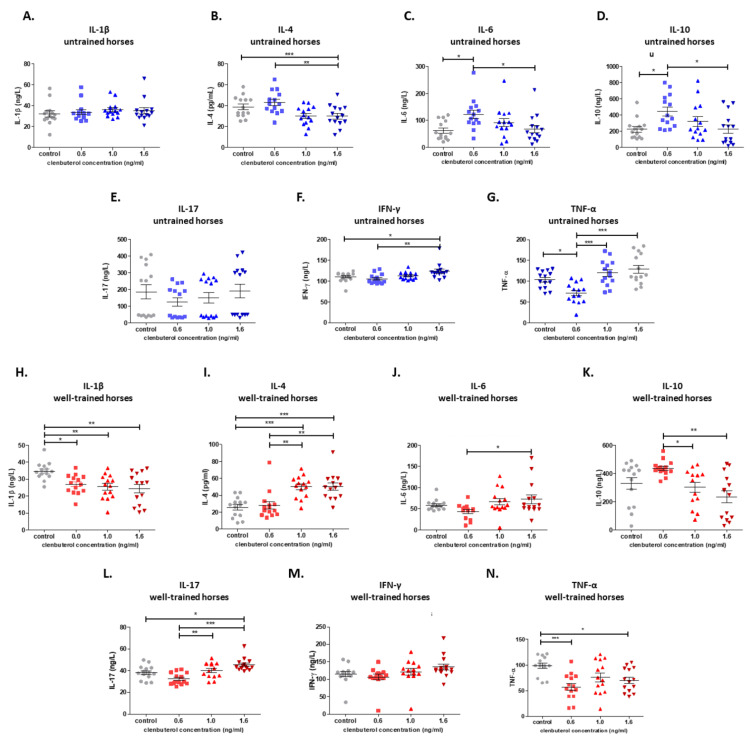
Graphic representation showing the cytokine concentrations: IL-1β (**A**,**H**), IL-4 (**B**,**I**), IL-6 (**C**,**J**), IL-10 (**D**,**K**), IL-17 (**E**,**L**), TNF-α (**F**,**M**), and IFN-γ (**G**,**N**) presented in the culture medium of control and clenbuterol-treated PBMCs obtained from untrained and well-trained horses. Each dot represents one individual horse (*n* = 28) and means ± SEM (standard error of the mean) are presented. The significance levels are: * *p* < 0.05; ** *p* < 0.01, and *** *p* < 0.001.

In contrast to untrained horses, in well-trained horses, a significantly decreased production of IL-1β under CLEN treatment at all concentration in comparison to non-treated cells was demonstrated, whereas no significant differences were found between treatments with different concentrations of CLEN (Figure 5H). The increased ability of these cells to secrete IL-4 (1–1.6 ng/mL) and IL-17 (1.6 ng/mL) and their decreased TNF-α production (1.6 ng/mL) were observed (Figure 5I,L,N). The influence of CLEN on IL-6, IL-10, and IFN-γ production was not detected. However, changes between cells treated with different drug concentrations were noticed only for IL-6 and IL-10 (and not for IFN-γ) (Figure 5J,K,M). Additionally, we observed difference in IL-4 and IL-17 production in cells treated with different CLEN concentrations (Figure 5I,L). 

## 4. Discussion

All findings suggest a potential health-threatening consequence of the illegal use of clenbuterol as a doping drug [12,13]. In fact, the reduction in the dysfunction of immune system cells caused by the prolonged consumption of CLEN may subvert immune surveillance. In addition, usage in combination with β2-agonists and corticosteroids has additive, synergistic, or opposite effects on inflammatory cells via modulation of the β2-AR gene’s expression [29]. During exercise, there is an increase in cortisol concentration, which is a beneficial response to physical activity because it plays an essential role in adaptation during exercise [30]. However, β2-ARs activation may block the beneficial training-induced enhancement of the cortisol response to acute exercise [27]. Thus, the study of β2-AR activation during physical activity is very important. To the authors’ best knowledge, this is the first study that confirms the dose-dependent effect on lymphocytes’ and monocytes’ phenotypes, functions, and reactive oxygen species production. 

### 4.1. Lymphocytes Phenotype and Proliferation

When comparing with other immune cell subtypes, β2-ARs expression is predominant in T cells in humans [31]. The influence on lymphocyte function is mostly connected with inhibited cytokine secretion and an altered cell-killing function [32]. Additionally, the cytotoxicity of lymphocytes is reduced by β2-ARs activation [33]. However, several studies have indicated that β-agonist therapy may actually worsen inflammation, for example, by restricting the T cells in immune organs [34]. Thus, β2-ARs activation has bidirectional effects on immune cells [35]. Additionally, physical activity alters the phenotype and reactivity of lymphocytes, which is duration-, intensity- and fitness-dependent in humans [36]. In horses, lymphocytes polarize to an anti-inflammatory phenotype in well-trained individuals, whereas in young horses, more pro-inflammatory reactions occur after acute short-duration exercise [23]. Thus, both exercise and β2-ARs activation influence immune cell activity. 

There has only been one study into the effect of chronic CLEN administration and physical training on leukocyte numbers in racing horses [15]. However, in that study, the post-exercise number of CD4+ lymphocytes was not significantly affected by CLEN administration in UT horses. Firstly, it may be influenced by the differences in drug concentration used, because the CLEN serum level was not measured in the previous study (dose 2.4 μg/kg per os). Moreover, CLEN has a greater influence on untrained than on previously trained animals [37], because the adrenergic responsiveness related to the β-ARs density reduction occurs with aging [38]. The high sensitivity of β2-ARs in young animals may lead to abolishing its effect by β1-AR activation, which enhances the pro-inflammatory reactions [39]. CLEN is a moderately selective β2-ARs agonist, but at higher doses, all classes of β-adrenoreceptors are activated [40]. Additionally, it was documented that the long-acting β2-agonists response is altered in a concentration-dependent manner [41].

This study showed increased lymphocytes’ proliferation obtained from race horses under CLEN treatment. However, there is a lack of data about the regulation of lymphocyte proliferation and the mechanism of this process under CLEN stimulation. However, it has been demonstrated that CLEN may enhance the proliferation of other various types of cells; for example, skeletal muscle cells [42]. CLEN may activate many signaling pathways such as Pi3K/Akt/mTOR, Erk, and Jak2/SOCS/STAT3 [43,44,45,46,47]. All these mechanisms positively regulate cell divisions and the survival of lymphocytes [48,49,50,51,52]. Thus, the same mechanisms are suspected to be involved in the regulation of lymphocyte proliferation after CLEN treatment. However, further extended research is needed.

### 4.2. Monocytes and Reactive Oxygen Species Production

Phagocytes such as monocytes are a critical component of the innate immune system. It is worth noting that these cells play an important role in the elimination of invading microorganisms by phagocytosis and killing. In humans, there are three subsets of blood monocytes: classical (CD14++CD16−) and intermediate cells (CD14+CD16+), both of which have pro-inflammatory properties, and non-classical (CD14+CD16++), which have anti-inflammatory activity [53]. In horses, because of the low availability of species-specific monoclonal antibodies, it was documented that CD14−MHCII+ are equivalent to human non-classical monocytes, while CD14+MHCII+ represent the intermediate and CD14+MHCII− are the classical monocytes [23,54]. 

Monocytes express high numbers of membrane β2-AR [29]. In humans, it is documented that β2-AR activation can exert either a stimulatory or inhibitory effect in immune cells [55]. Monocytes β2-AR stimulation favors an anti-inflammatory response in humans [56]. The CLEN inhibition of monocyte differentiation to dendritic cells was confirmed [57]. Furthermore, the elevation of intracellular cAMP stimulated by β2-AR activation inhibits cell adhesion and chemotaxis [58]. Additionally, in horses, CLEN treatment directly influences the modulation of the early inflammatory response. β2-AR activation results in a reduction in pro-inflammatory cytokine production in equine airway macrophages, and a reduction in leukocyte influx in the bronchoalveolar space [59]. 

In the previous study, CLEN administration reduced the number of monocytes after exercise in horses [15]. However, no monocyte phenotype and function was evaluated. In the present study, there was no change in the post-exercise monocyte populations after CLEN administration in WT horses. This may be connected with conditioning and receptor desensitization, which was documented in human monocytes [60]. On the other hand, CLEN’s influence was dose-depended in UT horses. The low dose of CLEN increased MHCII+CD14−, while a decrease in MHCII−CD14+ monocytes was observed. Thus, the higher doses failed to stimulate the monocyte polarization to the anti-inflammatory phenotype in inexperienced horses. Thus, in UT horses, CLEN administration at high doses may stimulate not only β2-ARs but also β1-AR. In humans, such activation leads to pro-inflammatory reactions in monocytes [60]. 

The effect of the β2-ARs stimulation of PBMCs on reactive oxygen species (ROS) production has not been determined yet. ROS production is one of the more prominent monocyte functions associated with intracellular killing, and it allows them to modulate the functions of other immune cells. In both groups, CLEN administration resulted in the inhibition of post-exercise ROS production by CD14+ cells. This is in line with other studies wherein the functional consequence of β-ARs activation was the inhibition of ROS production by human monocytes [61,62]. 

In addition, it was documented that CLEN reduces oxidative stress in the whole organism, but only superoxide dismutase (SOD) and malondialdehyde (MDA) serum levels were measured [63].

### 4.3. Cytokines

β2-ARs activation may also affect immune function through the modulation of cytokine production. In humans, there have been several reports of β2-agonist inhibition of cytokine release, such as TNF-α [29]. In the present study, pro-inflammatory cytokine (such as IL-1β and TNF-α) post-exercise production was inhibited by β2-ARs activation in WT, whereas in UT horses no differences were found regarding IL-1β. This is in line with humans studies, in which CLEN is a very potent inhibitor of the lipopolysaccharide (LPS)-induced release of TNF-α and IL-1β [8,64]. In horses, the effects of this drug have only been shown on clinical symptoms and leukocyte responses, which is mainly consistent with the production of TNFα and IL-6 after LPS stimulation [9]. In another study, CLEN at high concentrations (>313.6 ng/mL [10^−6^ M]) inhibited TNFα production in response to peptidoglycans (PG) and lipoteichoic acid (LTA), which are released from both Gram-positive and Gram-negative bacteria [14]. However, in the present study, high CLEN doses resulted in the stimulation of INFγ synthesis in inexperienced horses, which may be connected with β1-ARs activation, which stimulates the pro-inflammatory cytokine production [39]. Interestingly, IL-17 production was stimulated by high doses of CLEN in WT horses. However, this may be connected with increased Th17 cells, which are the lineage of effector CD4+ T cells [65]. As mentioned earlier, the percentage of CD4+ cells was the most affected by the high dose of CLEN. 

CLEN’s anti-inflammatory effect may also be achieved by the increased production of IL-4, IL-6 and IL-10. IL-10’s main biological function seems to be to limit and terminate the inflammatory responses, block pro-inflammatory cytokine secretion and regulate the differentiation and proliferation of several immune cells, such as T cells, B cells, natural killer cells (NK), antigen-presenting cells, mast cells, and granulocytes [66], whereas IL-4 and IL-6 may have both pro- and anti-inflammatory actions [66,67]. In the present study, the post-exercise increased production of IL-6 and IL-10 cytokines was confirmed only in UT horses after low CLEN dose stimulation. This is in line with another study, in which the LPS-induced expression of IL-10 mRNA in the PBMCs from horses with airway disease was altered after CLEN administration [68]. In WT horses, IL-6 and IL10 levels were dose-dependent. However, this did not influence the post-exercise level of those cytokines. However, the IL-4 production was increased after high-dose CLEN administration in WT horses, whereas in UT it was decreased after CLEN administration. Thus, the precise function of β2-ARs in cytokine production is still open to debate.

### 4.4. Limitations

Based on good ethical practices in performing experiments in animal models, the study was not performed in vivo, which is the main limitation of the study. In addition, animal investigations are limited in race horses during training season because of doping control. Thus, in vitro data are therefore essential in gaining basic information that can be translated in vivo. In addition, actions had to be taken to obtain as accurate results as possible during the experiment’s design. 

## 5. Conclusions

The results of this study suggest that a dose-dependent anti-inflammatory reaction occurred after CLEN-stimulation in exercised horses. However, in untrained animals, higher doses resulted in pro-inflammatory cell activity. Thus, the immune system in untrained horses in race training is more sensitive to drug abuse. The doping control should always be rigorous to ensure the animals’ welfare. Besides this, reducing leukocyte activation, pro-inflammatory cytokine production, and tissue damage by β2-ARs activation may be a novel therapeutic target in equine species. The results of these studies are very important for modern pharmacotherapy in human and veterinary medicine.

## Figures and Tables

**Table 1:** List of monoclonal antibodies used for labeling peripheral blood mononuclear cells (PBMCs) for flow cytometry.

Antibody	Clone; Dilution	Source	Target Cell
CD4:PE	CVS4; 1:10	BioRad, California, USA	Lymphocytes
CD8:FITC	CVS21; 1:10	BioRad, California, USA	Lymphocytes
CD5:PE	CVS5; 1:10	BioRad, California, USA	Lymphocytes
CD14:AF405	433423; 1:10	R&D Systems, Minnesota, USA	Monocytes
MHCII:FITC	CVS20; 1:20	BioRad, California, USA	Monocytes
FoxP3:APC	FJK-16s; 1:10	Life Technologies, Bleiswijk, Netherland;	Lymphocytes

## Data Availability

All data available from corresponding author.

## References

[1] Robinson N.E. (2000). Clenbuterol and the horse. Annu. Conv. AAEP.

[2] Couet W., Girault J., Reigner B.G., Ingrand I., Bizouard J., Acerbi D., Chiesi P., Fourtillan J.B. (1989). Steady-State Bioavailability and Pharmacokinetics of Ambroxol and Clenbuterol Administered Alone and Combined in a New Oral Formulation. Int. J. Clin. Pharmacol. Ther. Toxicol..

[3] Lynch G.S., Ryall J.G. (2008). Role of Beta-Adrenoceptor Signaling in Skeletal Muscle: Implications for Muscle Wasting and Disease. Physio.l Rev..

[4] Törneke K. (1999). β-Adrenoceptors in Equine Trachea and Heart. Vet. Res. Commun..

[5] Bijman J., Quinton P.M. (1984). Predominantly Beta-Adrenergic Control of Equine Sweating. Am. J. Physiol..

[6] Sanders V.M. (2012). The Beta2-Adrenergic Receptor on T and B Lymphocytes: Do We Understand It Yet?. Brain Behav. Immun..

[7] Chavan S.S., Tracey K.J. (2017). Essential Neuroscience in Immunology. J. Immunol..

[8] Izeboud C.A., Monshouwer M., van Miert A.S., Witkamp R.F. (1999). The Beta-Adrenoceptor Agonist Clenbuterol Is a Potent Inhibitor of the LPS-Induced Production of TNF-Alpha and IL-6 in Vitro and in Vivo. Inflamm. Res..

[9] Cudmore L.A., Muurlink T., Whittem T., Bailey S.R. (2013). Effects of Oral Clenbuterol on the Clinical and Inflammatory Response to Endotoxaemia in the Horse. Res. Vet. Sci..

[10] Spiller H.A., James K.J., Scholzen S., Borys D.J. (2013). A Descriptive Study of Adverse Events from Clenbuterol Misuse and Abuse for Weight Loss and Bodybuilding. Subst. Abus..

[11] Parr M.K., Koehler K., Geyer H., Guddat S., Schänzer W. (2008). Clenbuterol Marketed as Dietary Supplement. Biomed. Chromatogr..

[12] Abdulredha W.S. (2019). Effect of Clenbuterol Using as Weight Loose on Liver Enzymes and Lipids Profile. Iraq Med. J..

[13] Li C., Adhikari B.K., Gao L., Zhang S., Liu Q., Wang Y., Sun J. (2018). Performance-Enhancing Drugs Abuse Caused Cardiomyopathy and Acute Hepatic Injury in a Young Bodybuilder. Am. J. Men’s Health.

[14] Cudmore L., Whittem T., Bailey S. (2015). Anti-Inflammatory Effects of Clenbuterol on Equine Leukocytes Stimulated Ex Vivo with Bacterial Toxins. Aust. Equine Vet..

[15] Malinowski K., Kearns C.F., Guirnalda P.D., Roegner V., McKeever K.H. (2004). Effect of Chronic Clenbuterol Administration and Exercise Training on Immune Function in Horses. J. Anim. Sci..

[16] Cavalié H., Lac G., Lebecque P., Chanteranne B., Davicco M.-J., Barlet J.-P. (2002). Influence of Clenbuterol on Bone Metabolism in Exercised or Sedentary Rats. J. Appl. Physiol..

[17] Momaya A., Fawal M., Estes R. (2015). Performance-Enhancing Substances in Sports: A Review of the Literature. Sports Med..

[18] Weiser B., Drape J. (2020). More Than Two Dozen Charged in Horse Racing Doping Scheme. The New York Times.

[19] (2018). Economic Impact of the U.S. Horse Industry.

[20] Wong J.K.Y., Wan T.S.M. (2014). Doping Control Analyses in Horseracing: A Clinician’s Guide. Vet. J..

[21] Kakanis M.W., Peake J., Brenu E.W., Simmonds M., Gray B., Hooper S.L., Marshall-Gradisnik S.M. (2010). The Open Window of Susceptibility to Infection after Acute Exercise in Healthy Young Male Elite Athletes. Exerc. Immunol. Rev..

[22] Wood J.L.N., Newton J.R., Chanter N., Mumford J.A. (2005). Association between Respiratory Disease and Bacterial and Viral Infections in British Racehorses. J. Clin. Microbiol..

[23] Witkowska-Piłaszewicz O., Pingwara R., Winnicka A. (2020). The Effect of Physical Training on Peripheral Blood Mononuclear Cell Ex Vivo Proliferation, Differentiation, Activity, and Reactive Oxygen Species Production in Racehorses. Antioxidants.

[24] Witkowska-Piłaszewicz O., Bąska P., Czopowicz M., Żmigrodzka M., Szarska E., Szczepaniak J., Nowak Z., Winnicka A., Cywińska A. (2019). Anti-Inflammatory State in Arabian Horses Introduced to the Endurance Training. Animals.

[25] Witkowska-Piłaszewicz O., Bąska P., Czopowicz M., Żmigrodzka M., Szczepaniak J., Szarska E., Winnicka A., Cywińska A. (2019). Changes in Serum Amyloid A (SAA) Concentration in Arabian Endurance Horses During First Training Season. Animals.

[26] Resolution on the Animals Protection Used for Scientific and Educational Purposes and the European Directive EU/2010/63-Art 1.2 (5) Ust. z Dnia 15 Stycznia 2015 r. o Ochronie Zwierząt Wykorzystywanych Do Celów Naukowych Lub Edukacyjnych, Dz.U.2018.0.1207. https://sip.lex.pl/akty-prawne/dzienniki-UE/ochrona-zwierzat-wykorzystywanych-do-celow-naukowych-67984065.

[27] Kearns C.F., McKeever K.H. (2009). Clenbuterol and the Horse Revisited. Vet. J..

[28] (1998). Ventipulmin® Syrup (Clenbuterol Hydrochloride) Freedom of Information Summary.

[29] Johnson M. (2002). Effects of Beta2-Agonists on Resident and Infiltrating Inflammatory Cells. J. Allergy Clin. Immunol..

[30] Witkowska-Piłaszewicz O., Grzędzicka J., Seń J., Czopowicz M., Żmigrodzka M., Winnicka A., Cywińska A., Carter C. (2021). Stress Response after Race and Endurance Training Sessions and Competitions in Arabian Horses. Prev. Vet. Med..

[31] Ross A.M., Lee C.S., Lutsep H., Clark W.M. (2018). The Influence of β-Adrenergic Receptor Kinase-1 on Stroke-Induced Immunodeficiency Syndrome. J. Cardiovasc. Nurs..

[32] Grailer J.J., Haggadone M.D., Sarma J.V., Zetoune F.S., Ward P.A. (2014). Induction of M2 Regulatory Macrophages through the β-Adrenergic Receptor with Protection during Endotoxemia and Acute Lung Injury. J. Innate Immun..

[33] De Lorenzo B.H.P., de Oliveira Marchioro L., Greco C.R., Suchecki D. (2015). Sleep-Deprivation Reduces NK Cell Number and Function Mediated by β-Adrenergic Signalling. Psychoneuroendocrinology.

[34] Tracey K.J. (2014). Lymphocyte Called Home: Β2-Adreneric Neurotransmission Confines T Cells to Lymph Nodes to Suppress Inflammation. J. Exp. Med..

[35] Wu L., Tai Y., Hu S., Zhang M., Wang R., Zhou W., Tao J., Han Y., Wang Q., Wei W. (2018). Bidirectional Role of Β2-Adrenergic Receptor in Autoimmune Diseases. Front. Pharmacol..

[36] Nieman D.C., Miller A.R., Henson D.A., Warren B.J., Gusewitch G., Johnson R.L., Davis J.M., Butterworth D.E., Herring J.L., Nehlsen-Cannarella S.L. (1994). Effect of High- versus Moderate-Intensity Exercise on Lymphocyte Subpopulations and Proliferative Response. Int. J. Sports Med..

[37] Murphy R.J.L., Béliveau L., Seburn K.L., Gardiner P.F. (1996). Clenbuterol Has a Greater Influence on Untrained than on Previously Trained Skeletal Muscle in Rats. Eur. J. Appl. Physiol..

[38] Ferrara N., Komici K., Corbi G., Pagano G., Furgi G., Rengo C., Femminella G.D., Leosco D., Bonaduce D. (2014). β-Adrenergic Receptor Responsiveness in Aging Heart and Clinical Implications. Front. Physiol..

[39] Grisanti L.A., Evanson J., Marchus E., Jorissen H., Woster A.P., DeKrey W., Sauter E.R., Combs C.K., Porter J.E. (2010). Pro-Inflammatory Responses in Human Monocytes Are Beta1-Adrenergic Receptor Subtype Dependent. Mol. Immunol..

[40] Willson C. (2009). XPharm: The Comprehensive Pharmacology Reference.

[41] Sekut L., Champion B.R., Page K., Menius J.A., Connolly K.M. (1995). Anti-Inflammatory Activity of Salmeterol: Down-Regulation of Cytokine Production. Clin. Exp. Immunol..

[42] Katoch S.S., Sharma K. (2004). Clenbuterol Treatment Stimulates Cell Proliferation in Denervated Chick Gastrocnemius Muscle. Indian J. Exp. Biol..

[43] McNamee E.N., Ryan K.M., Griffin E.W., González-Reyes R.E., Ryan K.J., Harkin A., Connor T.J. (2010). Noradrenaline Acting at Central Beta-Adrenoceptors Induces Interleukin-10 and Suppressor of Cytokine Signaling-3 Expression in Rat Brain: Implications for Neurodegeneration. Brain Behav. Immun..

[44] Zhang Q., Xiang J., Wang X., Liu H., Hu B., Feng M., Fu Q. (2010). Β2-Adrenoceptor Agonist Clenbuterol Reduces Infarct Size and Myocardial Apoptosis after Myocardial Ischaemia/Reperfusion in Anaesthetized Rats. Br. J. Pharmacol..

[45] Kline W.O., Panaro F.J., Yang H., Bodine S.C. (2007). Rapamycin Inhibits the Growth and Muscle-Sparing Effects of Clenbuterol. J. Appl. Physiol..

[46] Milioto C., Malena A., Maino E., Polanco M.J., Marchioretti C., Borgia D., Pereira M.G., Blaauw B., Lieberman A.P., Venturini R. (2017). Beta-Agonist Stimulation Ameliorates the Phenotype of Spinal and Bulbar Muscular Atrophy Mice and Patient-Derived Myotubes. Sci. Rep..

[47] Sumi K., Higashi S., Natsume M., Kawahata K., Nakazato K. (2014). Temporal Changes in ERK Phosphorylation Are Harmonious with 4E-BP1, but Not P70S6K, during Clenbuterol-Induced Hypertrophy in the Rat Gastrocnemius. Appl. Physiol. Nutr. Metab..

[48] Yu C.-R., Dambuza I.M., Lee Y.-J., Frank G.M., Egwuagu C.E. (2013). STAT3 Regulates Proliferation and Survival of CD8+ T Cells: Enhances Effector Responses to HSV-1 Infection, and Inhibits IL-10+ Regulatory CD8+ T Cells in Autoimmune Uveitis. Mediat. Inflamm..

[49] So L., Fruman D.A. (2012). PI3K Signaling in B and T Lymphocytes: New Developments and Therapeutic Advances. Biochem. J..

[50] Zeng H., Chi H. (2013). MTOR and Lymphocyte Cell Metabolism. Curr. Opin. Immunol..

[51] Xue L., Chiang L., Kang C., Winoto A. (2008). The Role of the PI3K-AKT Kinase Pathway in T Cell Development beyond the β Checkpoint. Eur. J. Immunol..

[52] D’Souza W.N., Chang C.-F., Fischer A.M., Li M., Hedrick S.M. (2008). The Erk2 MAPK Regulates CD8 T Cell Proliferation and Survival. J. Immunol..

[53] Frellstedt L., Waldschmidt I., Gosset P., Desmet C., Pirottin D., Bureau F., Farnir F., Franck T., Dupuis-Tricaud M.-C., Lekeux P. (2014). Training Modifies Innate Immune Responses in Blood Monocytes and in Pulmonary Alveolar Macrophages. Am. J. Respir. Cell Mol. Biol..

[54] Rzepecka A., Żmigrodzka M., Witkowska-Piłaszewicz O., Cywińska A., Winnicka A. (2019). CD4 and MHCII Phenotypic Variability of Peripheral Blood Monocytes in Dogs. PLoS ONE.

[55] Wu H., Chen J., Song S., Yuan P., Liu L., Zhang Y., Zhou A., Chang Y., Zhang L., Wei W. (2016). Β2-Adrenoceptor Signaling Reduction in Dendritic Cells Is Involved in the Inflammatory Response in Adjuvant-Induced Arthritic Rats. Sci. Rep..

[56] Elenkov I.J., Wilder R.L., Chrousos G.P., Vizi E.S. (2000). The Sympathetic Nerve—An Integrative Interface between Two Supersystems: The Brain and the Immune System. Pharmacol. Rev..

[57] Giordani L., Cuzziol N., Del Pinto T., Sanchez M., Maccari S., Massimi A., Pietraforte D., Viora M. (2015). Β2-Agonist Clenbuterol Hinders Human Monocyte Differentiation into Dendritic Cells. Exp. Cell Res..

[58] Condino-Neto A., Vilela M.M., Cambiucci E.C., Ribeiro J.D., Guglielmi A.A., Magna L.A., De Nucci G. (1991). Theophylline Therapy Inhibits Neutrophil and Mononuclear Cell Chemotaxis from Chronic Asthmatic Children. Br. J. Clin. Pharmacol..

[59] Read S.A., Wijaya R., Ramezani-Moghadam M., Tay E., Schibeci S., Liddle C., Lam V.W.T., Yuen L., Douglas M.W., Booth D. (2019). Macrophage Coordination of the Interferon Lambda Immune Response. Front. Immunol..

[60] Lorton D., Bellinger D.L. (2015). Molecular Mechanisms Underlying β-Adrenergic Receptor-Mediated Cross-Talk between Sympathetic Neurons and Immune Cells. Int. J. Mol. Sci..

[61] Scanzano A., Schembri L., Rasini E., Luini A., Dallatorre J., Legnaro M., Bombelli R., Congiu T., Cosentino M., Marino F. (2015). Adrenergic Modulation of Migration, CD11b and CD18 Expression, ROS and Interleukin-8 Production by Human Polymorphonuclear Leukocytes. Inflamm. Res..

[62] De Angelis E., Pecoraro M., Rusciano M., Ciccarelli M., Popolo A. (2019). Cross-Talk between Neurohormonal Pathways and the Immune System in Heart Failure: A Review of the Literature. Int. J. Mol. Sci..

[63] Liu P., Xiang J., Zhao L., Yang L., Hu B., Fu Q. (2008). Effect of Β2-Adrenergic Agonist Clenbuterol on Ischemia/Reperfusion Injury in Isolated Rat Hearts and Cardiomyocyte Apoptosis Induced by Hydrogen Peroxide. Acta Pharmacol. Sin..

[64] Yoshimura T., Kurita C., Nagao T., Usami E., Nakao T., Watanabe S., Kobayashi J., Yamazaki F., Tanaka H., Inagaki N. (1997). Inhibition of Tumor Necrosis Factor-Alpha and Interleukin-1-Beta Production by Beta-Adrenoceptor Agonists from Lipopolysaccharide-Stimulated Human Peripheral Blood Mononuclear Cells. Pharmacology.

[65] Manni M., Granstein R.D., Maestroni G. (2011). Β2-Adrenergic Agonists Bias TLR-2 and NOD2 Activated Dendritic Cells towards Inducing an IL-17 Immune Response. Cytokine.

[66] Asadullah K., Sterry W., Volk H.D. (2003). Interleukin-10 Therapy—Review of a New Approach. Pharmacol. Rev..

[67] Reihmane D., Dela F. (2014). Interleukin-6: Possible Biological Roles during Exercise. Eur. J. Sport Sci..

[68] van den Hoven R., Duvigneau J.C., Hartl R.T., Gemeiner M. (2006). Clenbuterol Affects the Expression of Messenger RNA for Interleukin 10 in Peripheral Leukocytes from Horses Challenged Intrabronchially with Lipopolysaccharides. Vet. Res. Commun..

